# Adaptations and Predispositions of Different Middle European Arthropod Taxa (Collembola, Araneae, Chilopoda, Diplopoda) to Flooding and Drought Conditions

**DOI:** 10.3390/ani2040564

**Published:** 2012-10-18

**Authors:** Michael Thomas Marx, Patrick Guhmann, Peter Decker

**Affiliations:** 1 Institute of Zoology, Johannes Gutenberg-University of Mainz, Department IV, Becherweg 13, 55128 Mainz, Germany; E-Mail: pguhmann@web.de; 2 Department of Soil Zoology, Senckenberg Museum of Natural History Görlitz, Edaphobase, P.O. Box 300154, 02806 Görlitz, Germany; E-Mail: peter.decker@senckenberg.de

**Keywords:** invertebrates, climate change, periodic flooding, aperiodic flooding, drought

## Abstract

**Simple Summary:**

This review summarizes adaptations and predispositions of different arthropod taxa (springtails, web spiders, millipedes and centipedes) to flood and drought conditions. The main focus sis directed to arthropod species, which are living in Middle European floodplain forests and wetlands, because of the fast change of flood and drought conditions in these habitats. Furthermore the effects of the predicted regional climate change like increasing aperiodic summer flooding and decreasing winter and spring floods are also discussed.

**Abstract:**

Floodplain forests and wetlands are amongst the most diverse and species rich habitats on earth. Arthropods are a key group for the high diversity pattern of these landscapes, due to the fact that the change between flooding and drought causes in different life cycles and in a variety of adaptations in the different taxa. The floodplain forests and wetlands of Central Amazonia are well investigated and over the last 50 years many adaptations of several hexapod, myriapod and arachnid orders were described. In contrast to Amazonia the Middle European floodplains were less investigated concerning the adaptations of arthropods to flood and drought conditions. This review summarizes the adaptations and predispositions of springtails, web spiders, millipedes and centipedes to the changeable flood and drought conditions of Middle European floodplain forests and wetlands. Furthermore the impact of regional climate change predictions like increasing aperiodic summer floods and the decrease of typical winter and spring floods are discussed in this article.

## 1. Introduction

Riparian zones like floodplain forests and wetlands are among the world’s most productive plant and animal habitats. First and foremost, this is a result of the special properties of this habitat, which are tied to the constant flux between flooding and drought. These fluctuating conditions lead to a highly dynamic biocenosis. In order to survive here, arthropods must possess specific adaptations that enable them to tolerate these “disturbances”. The adaptations of arthropods in the floodplain forests of the Amazon have been well studied (reviewed in [1,2,3,4]). In one region, in which high water periods triggered by the rainy season occur each year and in which the water level climbs relatively slowly, the animals have adapted to these conditions over the past several million years. It can be assumed that these adaptations involve “true” adaptations that have arisen over the course of evolutionary time. In contrast to the Amazon wetland system, the floodplain forests of Central Europe have existed only since the end of the last glacial period. Here too, arthropods have diverse adaptations to the sometimes highly dynamic conditions. Some invertebrates show physiological resistance against inundation, phenologies in rhythm of the regular seasonal water level fluctuations and high dispersal ability as well as migration [2]. However, these adaptations presumably did not evolve over the course of the settlement of these habitats. Instead, they are the result of previously developed predispositions for which these specialized habitats presented a selective advantage [5]. Most species in middle European riparian habitats survive inundation using a “risk strategy”. This means the combination of high reproduction rates, dispersal and reimmigration following flood events [5]. This “r-strategy” results in high population densities in the spring period when regular floods recede. But extreme summer drought and aperiodic summer floods can cause drastic decreases in abundance and comprise the risk to disappear from these habitats. But strong dispersal ability, particularly by emigration to adjacent upland areas, increases the possibility to a later immigration of the flooded habitats. This strategy is found in many pioneer colonizers of high disturbed, immature or not yet developed ecosystems [5]. 

The majority of studies of Central European riparian zones have explored the effects of flooding on arthropods [6,7,8,9,10,11,12,13,14,15,16]. In contrast, studies of the effects of drought in these habitats are relatively rare [17,18], most likely because of the problem of the definition of drought in a hydrological system [19]. Humphries and Baldwin therefore hypothesized that in the future, different researchers should define the term “drought” in a hydrological sense and in a manner specific to their own field of research [20]. In this way, the greatest challenges are generally not in the droughts, but rather in the so-called “anti-droughts” [19]. Anti-droughts are the effects that are manifested by a change in the seasonality of the flooding events in a distinctly aquatic ecosystem. After prolonged dry periods or during a normal drought in a riparian zone, an aperiodic flooding event would likely have catastrophic consequences for the animal species living there. Anthropogenic encroachment in fluvial ecosystems has disrupted the delicate dynamics of the riparian regions of Central Europe. The consequences include the lowering of ground water levels, which has led to the disappearance of the natural flooding periods and hence to the desiccation of the riparian forests [21]. At the same time, an increase in the frequency of heavy rainfall events, particularly during the summer months, has increased the risk of aperiodic flooding in these regions. In association with the increased drought conditions, these floodings have devastating effects on the ecological communities in the riparian forests that remain. 

The effects of climate change on northern and Central Europe and regionally are the subject of several ongoing studies [22,23,24,25,26,27,28,29]. The various climate models used in these studies have predicted drier summers and milder, rainier winters [22,25,28,29,30,31]. The current temperature trends might indicate that the model predictions are more or less accurate. Over the past 30 years in Germany, 24 have turned out warmer than expected (source: German National Meteorological Service). Moreover, in the 130-year period of weather recordkeeping by the “German National Meteorological Service”, the 5 warmest years have occurred within the past 30 years. This has wide-ranging consequences especially for riverine ecosystems in Central Europe. The river Rhine, one of the largest rivers of Central Europe as well as for the riparian zones along it, will feature strong changes in the drainage regime. In comparison to former decades the length of the snow cover period is approximately 30–50% shorter in the lower and middle altitudes and shows an overall negative trend. The snow fall periods have similarly decreased [32]. Water, stored as snow throughout the winter and which leads to periodic flooding in the spring, flow off already in the winter months due to the higher temperatures. Furthermore, patterns of moist weather conditions with great rainfall quantities occur, increasing water levels even further [32,33,34]. However, this water level is generally not sufficient to achieve the previous complete flooding in the existing riparian zones and floodplain forests of the northern areas of the Upper Rhine. Within Germany, the Upper Rhine is one of the areas most affected by climate change [35]. In this region, the riparian habitats, typically showing a small scale mosaic of hillocks and depressions resulting in frequent and long-lasting to rare and short inundations, will suffer from increased drought conditions in the future. After the extreme drought of the summer 2003 no regular winter or spring flood occurred for the next nine years in the riparian zones of the Northern Upper Rhine Valley. Only three short-term partial floods took place in this timescale, which were caused mainly by seepage water. In past decades such partial floods occur often one to two times per year. If the flooding continues to fail for several years in a row, then these regions may lose their typical riparian character [36]. The diverse wet-dry niches and resources will vanish and may bring about a structural loss of well-adapted species.

Since the year 2000 different working groups of the Johannes Gutenberg-University of Mainz investigate several ecosystems of the Northern Upper Rhine Valley. One of the main topics of these investigations is to find out survival strategies of different arthropod groups in the riparian zones along the river Rhine and how these taxa cope with the increasing number of extreme events like extreme drought and aperiodic flooding. To our knowledge there is a lack of information about the effects of extreme drought and aperiodic flooding on different arthropod taxa in middle European riparian zones. This literature review provides an overview of the strategies used by various carnivorous and detritivorous arthropod taxa during regular periods of flooding and drought as well as aperiodic flooding. These taxa include the springtails, spiders, millipedes and centipedes, which are some of the main investigated arthropod groups of the Northern Upper Rhine Valley.

## 2. Collembola (Springtails)

Springtails inhabit every soil layer at very high densities and can even be found at the surface in great numbers, making them a key group among the soil arthropods. Over their long evolutionary history (the earliest date back to approximately 400 million years ago [37,38,39]), they have been able to colonize not only the deepest and uppermost levels of the soil, but have also populated plants and trees, the surface of water and other specialized and sometimes extreme habitats (e.g., deserts, the Arctic and the Antarctic) [40,41,42,43,44,45,46,47]. As a whole, this makes the Collembola one of the most ecologically diverse arthropod groups [48]. They are among the most important representatives of the soil food web [49] and according to Russell *et al.* [50], this order responds quite flexibly to disturbances to their habitats. 

There are several studies of the effects of flooding on the Collembola communities of riparian forests [50,51,52,53,54,55,56,57]. In particular, the work of Russell *et al.* [50] should be highlighted, in which they propose a new ecological classification of the Collembola at various study sites of the Upper Rhine based on their resistance to flooding. This subclassification might be a very useful tool for ecologists concerned with the classification of communities in flood areas. However, more study of the different species of Collembola is required. Furthermore, studies comparing this region with others comprised of different Collembola species should be conducted.

### 2.1. Flood Adaptations and Predispositions

**Figure 1:**
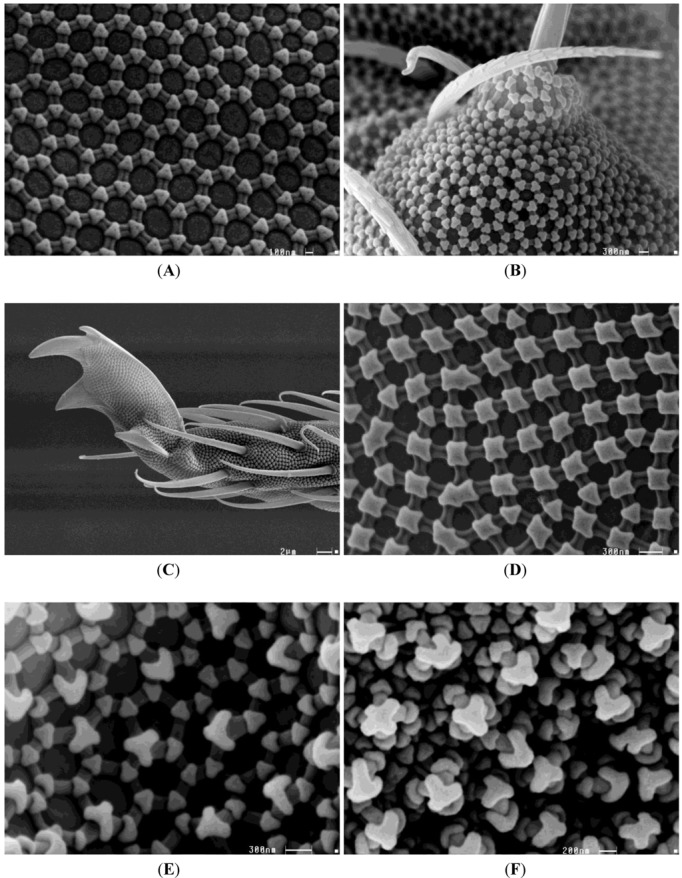
Different skin patterns of three collembolan families. (**A**,**B**) Entomobryidae: Typical honeycomb pattern made up of hexagonal granular units composed of microtubercles. (**C**,**D**) Isotomidae: More quadringular structure of the units. Note the uncovered regions of the furcal spines (C). (**E**,**F**) Onychiuridae: Secondary structure made of simple macrotubercles (E) and more complex macrotubercles (F). Bars: 100 nm (B), 200 nm (F), 300 nm (A,D,E), 2 µm (C).^ ©^ Stephan Borensztajn.

Among the Collembola, there are numerous adaptations to life on or in water. These adaptations include morphological, physiological and behavioral traits. Morphological adaptations include special structures on the surface cuticle that inhibit descent below the water surface. The basic model of the Collembola epicuticular structure resembles a honeycomb pattern made up of hexagonal granular units composed of microtubercles (Figure 1) [58,59,60]. 

This basic structure can vary among different species [61]. In addition to these microtubercles, some species have larger “warty” bumps called macrotubercles. Most forms that inhabit the ground (euedaphic species) have a relatively bare, hairless cuticle with hydrophobic properties that are strengthened by the formation of macrotubercles [62]. Ghiradella and Radigan [63] dyed the cuticles of *Tomocerus* (=*Pogonognathellus*) *flavescens* (Tullberg, 1871) (Tomoceridae) with lanthanum to demonstrate the presence of a hydrophobic lipid layer on the epicuticle. This species is an epedaphic form that inhabits the surface layer of the soil. The special composition of the surface structures in conjunction with the hydrophobic lipid layer likely helps to provide non-wettability of the cuticle. Furthermore the negative overhang causing a negative curvature in the profile of the ridges and tubercles are revealed as a highly effective design principle of collembolan skin [59]. Helbig and colleagues showed that due to this negative curvature an energy barrier must be overcome by the advancing liquid phase before wetting becomes irreversible even for liquids with very low surface tension [59]. This cuticular surface pattern is characterized as a structural plastron. A structural plastron is defined as any water-repellent structure of terrestrial and aquatic arthropods that allows air-breathing arthropods to use their respiration system under water. This consists of a thin air layer with a thickness of a few micrometer around the entire body (cuticular-breather; Collembola) or over certain body parts connected with the stigmata (tracheal breathers) [60,64]. In many Collembola species, these properties lead to a passive movement along the drift lines of water bodies. This drift may also aid their distribution along the waterway. Griegel was able to extract a total of six species of Collembola from samples of drift line water from the lower Oder valley, as well as an additional four species from the alluvial substrate [52]. Coulson *et al.* [65] and Moore [66] describe the possibility that passive drift is responsible for a variety of Collembola species across large geographical barriers and even as far as different arctic zones. The results of Coulson and Birkemoe are instrumental in demonstrating this possibility, because they showed that some species are able to survive more than four years at −22 °C [67]. Thus, movement can occur in a frozen state in pack ice. Fridriksson [68] studied a South Coast Island that appeared as a result of volcanic activity in 1963. After ten years, six species of Collembola could already be documented. Spread in the air or by means of different birds cannot be ruled out in this study, although passive drift across the ocean is the most parsimonious explanation for the colonization of this habitat by Collembola [65]. One of the advantages of passive drifting in middle European floodplains is the possibility of a quick resilience after regular and aperiodic flood events. But the individual number of springtail species which are able to survive the drift decrease strongly with increasing distances and time. Due to this fact passive drifting can be stated as a risk strategy for many species of the collembolan community. Other morphological adaptations for life on the surface of water include widening of various segments of the furca (ventral forked abdominal appendage—springing organ) that enables jumping on the water’s surface and provides special hydrophobic areas to the tibiotarsal region. Palissa’s book on epineustic Collembola provides a good overview of the various morphological design adaptations in different epineustic species of Collembola [46].

Physiological adaptations in Collembola species inhabiting the soil include a metabolic shift under anaerobic conditions (anoxia) [69]. For example, *Folsomia candida* Willem, 1902 (Isotomidae) have distinctly elevated lactate levels following artificially induced anoxia [9,70,71]. In addition, this species has been shown to have an increased heart rate in oxygen-deprived environments (hypoxia). The adaptive change in blood circulation should maintain the partial pressure between the medium, the blood and the tissue [72]. With this adaptation, individuals of *F. candida* can exploit extremely low partial pressures of oxygen down to 6,666 Pa for respiration. In *Anurida maritima* (Guérin-Méneville, 1836) (Neanuridae), the exploitable partial pressure of oxygen is even lower [73]. This species lives in the tidal zones and thus endures periodic flooding from ebbs and tides. At low tides, these animals feed on fine substrates such as the algae and suspended sediments of the surface. With increasing flooding, individuals gather in aggregations (“nests”) under stones in order to survive the flooded period in a common air pocket [74]. In such extreme conditions, making use of very low partial pressures of oxygen (as low as 1,000 Pa) can be very advantageous. A critical value for the animals could not be achieved experimentally by Zinkler *et al.* [73]. However, at 1,000 Pa, there was already a reduced but still regulated oxygen uptake.

The soil biocenosis in the flood zones of the Eder dam in Germany that undergo annual drought periods was investigated by Tamm [13,14] and Tamm *et al.* [16] for endangered specialist communities or opportunistic colonizers from the immediate surroundings. They documented a zoological community in the Eder dam region that is similar to those that are typical of forest-free riparian zones in Central Europe that have recently dried out. Five species of Collembola were found in the soil surface layer (*Isotoma viridis* Bourlet, 1839, *Anurida tullbergi* (Schött, 1891), *Sminthurides malmgreni* (Tullberg, 1876), *Sminthurinus aureus* (Lubbock, 1862) and *Sminthurus nigromaculatus* (Linnaeus, 1758)), which accounted for 99.9% of all Collembola sampled. These species are characterized by the ability to survive a flood period as eggs. The larvae hatch shortly after the flooded habitat dries out and are able to exclusively colonize the area. This ability has been demonstrated by taking underwater sediment samples and then “incubating” the Collembola eggs in a climate-controlled cabinet [14,15]. Even the species *Isotomiella minor* (Schäffer, 1896) (Isotomidae) and several other Symphypleona species, survival in the egg stage over long periods of flooding has been documented [75,76]. Some species of Collembola have the ability to survive for short periods in water (semiaquatic lifestyles). In these species, embryonic and post-embryonic development as well as the first molt, have been shown to take place under water [77]. 

Beck [78] demonstrated that adults of large species of epigeal Collembola (Entomobryomorpha) avoid flood period and there is a mass appearance on the shores. However, this phenomenon was studied in the slowly increasing water levels in the Amazon region. In cases where flooding occurs rapidly, springtails usually cannot actively escape and instead drift passively because of the non-wettability of their cuticle [58,62,63]. However small scale escape strategies like vertical trunk migration during the flood or remigration from non-flooded refugees after the flood were demonstrated [9].

### 2.2. Drought Adaptations and Predispositions

In contrast to the large number of studies of the adaptation of Collembola to flooding, there are relatively few that have examined adaptation to prolonged periods of drought [79,80,81]. On one hand, this deficit can be explained on the basis of the lifestyle of most Collembola species, which is closely related to the presence of water. On the other hand, habitats which require a high degree of drought resistance in Collembola are either difficult to access or can only be explored at great expense. For example, these include desert areas or the core areas of trees, which are sometimes subject to great fluctuations in water availability. 

Greenslade [43] formulated the following five protective mechanisms against desiccation for the species of Collembola in desert environments. (1) Tolerance to high temperatures and saturation deficits by means of morphological and physiological adaptations. (2) Reduction of the risk of desiccation by means of behavioral changes. (3) Dessication-resistent eggs and very short life cycles. (4) The potential to survive poor conditions by means of anhydrobiosis and ecomorphosis (both terms are described later in this section). (5) Colonization of residual moist surfaces in desert habitats. Similar adaptations against desiccation are also likely to exist in Collembola species in other regions and might also be true for drought periods in floodplains. One example of an adaptation against desiccation in *Entomobrya nivalis* (Linnaeus, 1758) and *Orchesella cincta* (Linnaeus, 1758) (both Entomobryidae) in Central European habitats is a thickened wax layer on the cuticle. Both species generally occur on trees at high densities and throughout the entire year [82,83,84]. 

*I. viridis* and *Isotomurus palustris* (Lubbock, 1870) (both Isotomidae) have both been shown to have remarkable adaptations to desiccation. During periods of increasing soil drought, individuals of these species undertake an intensive feeding bout and then retreat to small soil caverns and seal them with feces [46,85,86,87]. They are able to stay in these caverns in a lethargic state to survive the dry period. The advantage of this drought stasis is likely reduced evaporation resulting from immobility. However, the animals will still die in severe drought conditions [85,86,87]. A real anhydrobiosis was shown for the species *Folsomides angularis* (Axelson, 1905), which is able to live in desert ecosystems of South Europe but also can be found in Central and Northern Europe [88]. The anhydrobiotic state helps the animals to have better tolerance not only to higher temperatures but also to very low temperatures [88,89]. It is now understood that the predispositions against drought and extreme cold have the same origins and did not evolve independently from each other [90,91,92,93]. Hinton [94] provides a powerful example of this. He examined larvae of the species *Polypedilum vanderplanki* Hinton, 1951 (Chironomidae, Diptera), which are known for an effective anhydrobiosis, for their resistance to cold. He cooled the animals in an anhydrobiotic state to −270 °C, although the distribution of this species means they never actually encounter temperatures below zero. 

Altered climatic conditions can also lead to striking morphological changes in some species of Collembola. Cassagnau [95] found that unfavorable moisture conditions led to the loss of the bothriotricha and a shortening of the abdominal macrochaetes in *I. palustris.* These modifications are known as ecomorphosis. According to Palissa [46] the development of ecomorphs is generally the result of unfavorable hygrothermal conditions.

Drought resistance in *F. candida* has been relatively well studied in recent years, because this Collembola species play a key role in ecotoxicological studies because of its parthenogenic (asexual) mode of reproduction [49,96]. In an experiment by Sjursen *et al.* [97], individuals of the species *F. candida* were exposed to varying intensities of drought conditions. With an experimental design with a brief pre-induced dryness, the results showed increased survival and longer activity stages. When this design was evaluated physiologically, there were lower myo-inositol values with sharply elevated trehalose levels. This result suggests that this species is capable of gradually adapting to increasing drought conditions. When drought conditions are rapidly induced for long term, the animals immediately enter an anhydrobiotic state with a significantly reduced survival rate. Hence, in natural environments Collembola likely experience greater physiological stress when faced with extreme and long term drought than with periodic or aperiodic flooding. In addition, the activity of Collembola is permanently disrupted as a result of the lower survival rates in the soil, which leads to significantly reduced rates of decomposition in the soil ecosystem. Pflug and Wolters [98] studied this effect in a field experiment and also found significant reductions in the abundance and diversity of the collembolan community. Similar results with respect to diversity and density of Collembola were found in a study of riparian forests near Heidenfahrt [54].

In another adaptation to drought, a significant change to the composition of phospholipids in the membrane of *Protaphorura armata* (Tullberg, 1869) (Onychiuridae) can be induced by drought conditions [99]. In a series of experiments, Holmstrup *et al.* [91] showed similar modifications in *F. candida*, which could counteract the effects of the changed moisture concentrations and altered water potential in dehydrated individuals. The importance of cuticle permeability, osmolyte production and specific body sizes for drought resistance is highlighted by Kaersgaard *et al.* [100]. Nevertheless, Bayley and Holmstrup [101] noted the need for further studies of the physiology of drought resistance in Collembola. Please see Table 1 for details. 

**Table 1:** Different type of adaptations of Collembola to periodic and aperiodic flooding as well as drought events.

Type of Adaptation	Periodical flood	Aperiodical flood	Drought
*Morphological Adaptation*	Hydrophobic properties of the cuticle (structural plastron); Passive drifting; Modifications of furca and legs (epineustic species: e.g., Poduridae and Sminthurididae)	Hydrophobic properties of the cuticle (structural plastron); Passive drifting	Thickened wax layer on the epicuticle surface (*E. nivalis*,*O. cincta*); Ecomorphosis (*I. palustris*); Decreasing of cuticular permeability (*P. armata*, *F. candida*)
*Physiological Adaptation*	Metabolic change (lactate) (*F. candida*); Metabolic depression (*F. candida, A. maritima*); Egg-diapause (*I. viridis*,*A. tullbergi*, *S. malmgreni*,*S. aureus*,*S. nigromaculatus*,*I. minor*, some symphypleonid species); Increasing heart rate; Use of extreme low O_2_ partial pressures (*F. candida*)	Metabolic change (lactate) (*F. candida*); Metabolic depression (*F. candida, A. maritima*); Semiaquatic life style (Hypogastruridae)	Metabolic depression (drought stasis) (*I. viridis*,*I. palustris*); Anhydrobiosis (*F. angularis*) Drought resistant eggs
*Behavioral and phenological Adaptation*	Remigration after flood events from non-flooded sites and trees; Epineustic life form (epineustic species: e.g., Poduridae and Sminthurididae)	Epineustic life form (epineustic species: e.g., Poduridae and Sminthurididae)	Behavioral changes; Migrating to wet refugees; Short life cycles

## 3. Araneae (Spiders)

In Central Europe, the floodplain forests that are characterized by a regular flooding regime are among the most diverse habitats when it comes to species richness and abundance of spiders. The highly diverse structure of the floodplain forests provides spiders that inhabit strictly defined microhabitats with a broad spectrum of ecological niches. Limiting factors include physical conditions such as temperature, light, humidity, wind and light intensity, as well as biological factors such as vegetation, availability of food, competition and predator pressure. Vegetation structure can be divided into four strata: ground level, field, shrub and tree/crown [102]. Each stratum has a unique microclimate, opportunities for retreat and predator-prey system. So they serve as key drivers for many different arthropod species. In Middle European floodplains regular and aperiodic flooding are the most important factors for the changing conditions. Ecological separation of different species of spiders is achieved not only by their different spatial distributions, but also by various modes of reproduction and periods of activity. Many species are able to utilize the same microhabitat because their main phase of activity is concentrated in different seasons or times of the day [103]. 

### 3.1. Flood Adaptations and Predispositions

However, species-rich settlement of the riparian forests of Central Europe can only take place by species of spiders that have previously developed predispositions which allow them to colonize areas that are subject to periodic flooding. Opportunistic species, especially canopy and dwarf spiders, (Linyphiidae), as well as wolf spiders (Lycosidae), intentionally immigrate to previously flooded riparian zones and make use of the loosened resources. These pioneer species have a dynamic settlement pattern and high reproductive capacity [104]. They do not have special morphological adaptations for high water. Aperiodic floodings during the summer therefore lead to significant population crashes [105]. However, heavy dispersal by the subsequent generation into the surrounding areas enables a re-colonization of the floodplain forest following a flooding event. Thus most pioneer species are not associated with specific habitats. These euryoecious species have no specific habitat preferences, but prefer disturbed and mostly open habitats. Sites with high intensity of disturbances are colonized first [106]. According to Siepe [107], in areas that are frequently flooded, euryoecious pioneer species, especially Linyphiidae, are found close to the water line, whereas the proportion of Lycosidae increases significantly as the frequency of flooding decreases. 

Wohlgemuth-von Reiche and Grube [108] described the aeronautic ballooning of many dwarf spiders (Micryphantinae) and juvenile Lycosidae as a special phenomenon associated with non-directional migration. The aeronautic ballooning also occur in many other spider families. The spiders climbed on wind-exposed sites (grasses, twigs, *etc.*) and cast a thread up into the blowing air masses [109]. The thread exposed to the wind currents is stronger than that from spiders protected underground and functions as a balloon [110]. Thus, using the tiny surface area-to-volume ratio, light spiders with long spinning threads can reach even the slightest upwinds in order to drift passively. According to Thomas and Jepson [111], the number of hours during which meteorological conditions are suitable for “ballooning” each day increases steadily from June until the end of August. The ideal horizontal wind velocity is 3 m/s. In these conditions, the ratio between vertical and horizontal wind currents is high and allows even larger spiders to lift off from the ground. The fall emigration and early summer immigration using this aeronautic technique protects the spiders from periodic winter floods and according to Weigmann & Wohlgemuth-von Reiche [5] it is the key predisposition of euryoecious species that allows them to settle in riparian zones like floodplain forests and wetlands. 

Wolf spiders of the genus Pardosa are pre-adapted to both winter and summer floods because of their phenology. These animals overwinter as subadults in a diapause during which no molts take place [112]. The lowered metabolic rates during the winter diapause allow these spiders to survive periodic winter floods in hiding spots with air pockets [113]. In the spring, they molt into adults and pairing takes place in March [109]. After hatching in April and until molting into subadults in August, the larvae exhibit a highly developed migratory behavior. According to Manderbach [114], the adults can actively retreat from advancing floodwater. After an aperiodic summer flood, in August there is both a second mating among wolf spiders [114], and the spring generation returns to the dry habitat [109]. 

In addition to these euryoecious examples of spiders, there are a good number of species in riparian zones that are stenotopic. These species are particularly dependent on shady and regularly flooded moist habitats. Although these species can survive floodings unharmed, their dominance decrease significantly as the flood intensity decreases [115]. However, most of these species do not have special morphological adaptations for flood resistance. Instead it is more likely that these animals escape advancing floodwater by migrating vertically on tree trunks and in the shrubbery and survive unharmed in these higher levels [116,117]. Only nursery-web spiders (Pisauridae) and a few wolf spiders have hydrophobic body hairs and the ability to move about using a synchronized motion of the legs (“rowing”) [118]. These hydrophobic hairs also allow different species of the wolf spider genus *Pirata* temporary diving and hunting below the water surface. They catch different mosquito and insect larvae, water slaters and small crustaceans by visual hunting [103]. But mainly the *Pirata*-species hunt on the water surface. One exception with a main foraging under water is the water spider *Argyroneta aquatica* (Clerck, 1757), which is able to pull air under the water’s surface by means of its body hairs and to build a diving bell [119]. These diving bells serve as structural plastrons. The ability of the saltmarsh lycosid *Arctosa fulvolineata* (Lucas, 1846) to survive submersion up to 34 hours (50% survival: 20 hours) show the possibility of a metabolic depression, which was called “hypoxic coma” by Pétillon *et al.* [120]. 

### 3.2. Drought Adaptations and Predispositions

The spider communities of Central Europe are predominantly composed of species that prefer open habitats. A large number are stenotopic inhabitants of warm sites with mosaic-like structure [121]. These habitats can be successfully colonized by spiders in high abundances since they have a variety of different morphological and behavioral adaptations to drought and heat. Like all arthropods, spiders are susceptible to high rates of evaporation because of their small body sizes and large surface area-to-volume ratios. Thus, one of the most important arthropod adaptations to drought is the reduction of transpiration via the cuticle and respiration [122]. The cuticle of spiders is similar in construction to that of insects [123,124]. The wax layer of the epicuticle is the chief barrier against water loss. It is therefore constructed especially effectively in thermo- and xerophilic species [125]. In these species, the waxy layer has a high density since the hydrocarbons of which it is composed are mostly made up of branched alkanes [126]. At temperatures beyond 40 °C, the wax layer of *Geolycosa godeffroyi* (L. Koch, 1865) loses its stability and becomes permeable to water [122,127]. Spiders are able to perceive changes in atmospheric humidity using hygroreceptive sensilla on the tarsal organ [128,129]. They respond to decreasing humidity by reducing the water content of the endocuticle and thus lower the overall permeability of their exoskeleton [130]. 

The respiration system of spiders shows also different predispositions for living in dry environments. Respiration in most spiders (Tracheospira) occurs via diffusion across the epithelium of book lungs and tubular trachea. Whereas oxygen taken up via the book lungs must be transported via the haemolymph to the organs, diffusion via the trachea occurs directly to the tissues requiring oxygen. With book lungs blocked, living individuals of the lycosid species *Pardosa amentata* (Clerck, 1757) (which survive well for 15 minutes exposures) show a decrease in rate of transpiration by about a third to a fifth at temperatures up to 40 °C, above which evaporation is much more rapid. Consequently, respiratory water loss represents about a third or less of total transpiration in this species [122]. Spiders that are active walkers with high oxygen demands thus have a highly branched tracheal system and reduced book lungs [131]. The efficacy of respiration via the tubular tracheal system means the spiders can have a comparatively lower respiratory surface and hence lose less water. For this reason, some authors believe that the development of a complex tracheal system is an adaptation for life in dry habitats [132,133,134]. A behavioral adaptation to avoid water loss through respiration is the closing of the book lung openings through spiracles [127]. The spiders can keep the lung atria closed during periods of high physical stress and enter a state of oxygen deficit. This deficit is then balanced during long rest periods by means of heavy ventilation through the book lungs [135]. Spiders minimize water loss via excretion by the formation of highly concentrated urine [136], and the water that is lost is recovered through feeding and drinking of capillary water [137]. Coxal organs are reduced in more highly developed spiders; in Mygalomorpha (bird spiders) these organs function only in the regulation of the haemolymph-ionic balance [138].

There exist also some adaptations in early developmental stages against drought. The majority of spiders envelop their eggs with silk cocoons in which the embryo develops and from which the nymph hatches. Depending on the species, the young spiders leave the egg cocoon shortly after the first molt or overwinter within the protection of the cocoon. Species that overwinter in the cocoon protect the larva from desiccation, while the cocoons of species that do not overwinter offer the eggs no protection from transpiration [139]. According to Austin [140], the cocoons of *Clubiona robusta* (L. Koch, 1873, Clubionidae, sac spiders) have a higher humidity than the surrounding air and prevent the eggs from drying out. Schaefer [141] experimentally demonstrated that the eggs of *Floronia bucculenta* (Clerck, 1757) (Linyphiidae) survive in the cocoon for 68 days at 32% relative humidity and 5 °C. Without cocoon, the eggs dry out in 37 days. In contrast, Austin and Anderson [142] showed that the cocoons of *Nephila edulis* (Labillardière, 1799) (Araneidae, orb-web spiders) offer no protection from desiccation. 

As poikilotherms, spiders are capable of actively regulating their body temperature by behavioral changes. Spiders that move actively avoid overheating by cooling off in shady spots [127]. Web-spinning spiders place their bodies parallel to the incident light and thus reduce the amount of their body’s surface that is exposed to sunlight [143]. Furthermore, they have reflective guanine crystals in the cuticle of their opisthosoma to protect them from overheating [103]. Please see Table 2 for details. 

**Table 2:** Different type of adaptations of Araneae to periodic and aperiodic flooding as well as drought events.

Type of Adaptation	Periodical flood	Aperiodical flood	Drought
*Morphological Adaptation*	Hydrophobic hairs allow movement on the water surface (“rowing”) (Lycosidae and Pisauridae)	Hydrophobic hairs allow movement on the water surface (“rowing”) (Lycosidae and Pisauridae)	Decreasing of cuticular permeability (*G. godeffroyi*); Low water loss because of the tracheae (Tracheospira); Cuticle with light reflecting guanine crystals
*Physiological Adaptation*	Low metabolic rate during hibernation (Lycosidae); Metabolic depression (*A. fulvolineata*)	--	Highly-concentrated urine; Transpiration reducing egg cocoons (*C. robusta*,*F. bucculenta*)
*Behavioral and phenological Adaptation*	Emigration in autumn an remigration in early summer (“ballooning”) (Micryphantinae, juvenile Lycosidae, Different other families); Horizontal and vertical migration	Horizontal and vertical migration; Second mating after summer flood (Lycosidae)	Closing of book lungs with spiracles during high physical load; Active body moving parallel to the incident sunlight (web-spinning spiders)

**Table 3:** Different type of adaptations of Diplopoda and Chilopoda to periodic and aperiodic flooding as well as drought events.

Type of Adaptation	Periodical flood	Aperiodical flood	Drought
*Morphological Adaptation*	Structural plastron	Structural plastron	Decreasing of the cuticular permeability (*O. ornatus*, *Thyropygus* sp.,*P. lagurus*)
*Physiological Adaptation*	Long submersion times in cold and oxygen-rich water (some polydesmid species)	--	--
*Behavioral and phenological Adaptation*	Hibernation in the egg-stage (*L. emarginatus, P. denticulatus*); Short developmental times (*L. emarginatus*)	--	Migration in deeper soil layers; Decreasing of transpiration due to curling (*Henia vesuviana*); Production of ootheca and water absorption by the eggs (*Archispirostreptus tumuliporus judaicus*)

## 4. Diplopoda and Chilopoda (Myriapoda: Millipedes and Centipedes)

### 4.1. Flood Adaptations and Predispositions

Because for a long term inhabiting of riparian habitats the ability to survive periods of flooding is an important factor, this characteristic also significantly influences the myriapod species composition found in these habitats. Adis [1] studied the different strategies used by terrestrial arthropods of the Central Amazon and was able to show that even myriapods have a variety of adaptations to flooding, such as a diapause within roots by *Ribautiella amazonica* Scheller, 1984 (Scolopendrellidae), small-scale changes of location along trunk in the arboreal species *Epinannolene arborea* Hoffmann, 1984 (Pseudoannolenidae), or the flood resistance in the eggs of the parthenogenic chilopod *Lamyctes* sp. (Henicopidae), which can develop into adults within six to eight weeks. *Gonographis adisi* Hoffmann, 1985 (Pyrgodesmidae) can survive in the upper stratus of the water for up to 11 months with the aid of plastron respiration and feeding on algae [144]. *Mestosoma hylaeicum* Jeekel, 1963 has a life cycle close adapted to flooding [145]. In Central Europe, however, regular winter and spring as well as aperiodic flooding events generally occur more irregularly and with a rapid increase in water level; these facts can have long-term consequences for the animals that live there. Longer periods of flooding may lead to almost complete decimation of the diplopod and chilopod populations [146]. With increasing frequency, intensity and duration of flooding events, the number of species decreases. The species *Lamyctes emarginatus* (Newport, 1844) is an example of a centipede with an opportunistic risk strategy [5]. A parthenogenic mode of reproduction and short development times of 6 to 12 weeks until adulthood and the ability of eggs to survive inundation periods and low temperature in winter allow this chilopod to permanently re-colonize areas that undergo regular and long-lasting floods [147,148]. 

Resistance to flooding is a key physiological predisposition. Diplopods that only colonize riparian forests in small numbers, survive submersion for a few hours up to several days [148,149,150]. In contrast, the flat-backed millipedes (Polydesmida) Polydesmus denticulatus (C.L. Koch, 1847) and *Brachydesmus superus* (Latzel, 1884) can survive under water for over 70 days [148] and *Polyzonium germanicum* Brandt, 1837 (Polyzoniidae) can survive up to 43 days [149]. In the one-year species *L. emarginatus*, only the eggs overwinter, and these remain capable of development even through several week-long winter or spring floodings and make it possible for the species to rapidly re-colonize the flooded areas [147,148].

However, these high levels of tolerance are only achieved in cool (4–10 °C) and oxygen-saturated water. The question remains, nonetheless, whether all populations achieve such high tolerance, since the selection pressure imposed by flooding can vary greatly amongst populations. In *P. denticulatus*, Zulka [148] was able to show that there was significantly higher tolerance to submersion in one population that was frequently exposed to flooding, compared to a population 3 km away that had not experienced a flood in 65 years. In water that is low in oxygen, for example warm, stagnant water or rising groundwater, even submersion-tolerant species survive only for a few hours [148]. A summer flood therefore has far more negative impacts on the existing coenosis compared to flooding events at cooler times of the year. There are no special phenological adaptations in the Central European riparian forests. The one-year species *L. emarginatus* is a good example of a relatively well adapted species that can survive in the egg stage through winter and summer flooding [5]. *P. denticulatus*, with its summer activity and reproductive period [151,152] and the short development time of the Polydesmides of Central Europe are also phenologically well-adapted to flooding events [148]. 

Directional and deliberate migrations have not yet been identified in diplopods and chilopods in areas subject to flooding. Active escape from a high water line is only possible to a limited extent, because of the slow mode of locomotion, which is particularly a factor for the diplopods. In addition, the various species also respond quite differently to rising water and show varying abilities to coordinate underwater, which does not necessarily always correlate well with their ties to riparian zones or submersion tolerance [148]. Diplopods have a low specific density, making them able to float on still water surfaces for only a few hours to days before they sink [150]. 

### 4.2. Drought Adaptations and Predispositions

In contrast to the insects, chilopods have no waxy cuticle and therefore always depend on a moist environment. Therefore the stronger heat periods which are predicted for Central Europe affect them in particular. Under unfavorable conditions such as heat, dryness or cold, they thus retreat to deeper soil levels or into crevice [153]. The geophilomorph species *Henia vesuviana* (Newport, 1845) decreases its transpiration rate during rest periods during which they roll themselves into a tight spiral [154]. The exoskeleton of diplopods is highly calcified and is permeable to water in large quantities, which is why most species are found only in moist environments or on moist microhabitats. Most species are active only at dusk, during the night or early mornings on the surface, when the atmospheric humidity is greater than it is during the day [155]. Diplopods have hygroreceptors (e.g., the organ of Tomosvary) on their sternites and largely photonegative behavior, which together allow the animals to seek out suitably moist shelters [156]. In both temperature and tropical regions, most species spend drier and warmer periods within the soil [157,158,159]. Mammal dens or termite hills or existing holes in the ground are also used to survive through periods of drought [159]. However, many species can also adapt to drier habitats. Animals that are not yet ready to reproduce construct thick-walled molt chambers during droughts. By wrapping themselves up, the animals reduce transpiration via the tracheal openings, since these cannot be actively closed by the animals [160]. Species that are well-adapted to drought, such as the American desert millipede *Orthoporus ornatus* (Girard, 1853) or the Indian millipede *Thyropygus* sp. (Spirostreptidae), reduce water loss across the exoskeleton by means of the epicuticle [156,161]. This layer is composed of lipoproteins and sudanophilic lipids, which unlike insects, do not lose stability even in very low or high temperatures and thus lead to uniform water loss. The production of ootheca from soil and plant material reduces the moisture gradients between the eggs or larva and the surroundings, depending on the water retention capacity of the materials used [162]. The eggs of *Archispirostreptus tumuliporus judaicus* (Attems, 1927) (Spirostreptidae) can actively absorb water from the surrounding environment (up to 10% of the egg’s weight) [163]. Eisenbeis and Wichard [58] showed that the bristly millipede *Polyxenus lagurus* (Linnaeus, 1758) (Polyxenidae) has very low loss from transpiration and an uptake of water vapor from the air. The animals were maintained in completely dry air (0% relative atmospheric humidity) for several days. The hourly reduction of water mass under these extreme conditions was less than 1%. In 98% relative humidity, absorption of water vapor was measured that was the equivalent of a weight increase of more than 3% per hour. The absorption took place between 5 and 6 AM. This matches the timing of dew formation, when a high environmental humidity is expected. These adaptations are very important in the habitat of *P. lagurus*, since this species must deal with a relatively dry environment in trunk habitats. Please see Table 3 for details. 

## 5. General Aspects of the Bioindication Value and Impacts of the Predicted Climate Change Scenario

Springtails demonstrate a wide range of different adaptations and predispositions to regular flood events. The species composition of undisturbed riparian zones and floodplain forests show high densities of different hygrophilic and hygrotolerant species after regular flooding. The bioindication value of these species can be estimated as very high. Russell and Griegel [55] detected a distinct succession of ecologically isovalent collembolan groups according to the flood intensity in the Upper Rhine Valley. Strong hygrophilic species like *Sminthurides* ssp., *Podura aquatica* and *Isotomurus palustris* are limited to frequently flooded sites, whereas hygrotolerant species like *Sminthurinus aureus* and some other *Isotomurus* species prefer less flood affected sites. Flood-intolerant species were only found in higher non-flooded sites or only after many weeks or months after inundation due to a slow migration to the flooded sites. The predicted climate change with prolonged drought periods and aperiodic flood events cause strong changes in the collembolan community of riparian habitats. In times of prolonged drought the well adapted hygrophilic and hygrotolerant species are replaced by more ubiqituous and opportunistic species [9]. The opportunistic species show mostly no effective adaptation or predisposition against flood. Thus after regular or aperiodic flooding the abundance of these species markedly decreases and the riparian habitats show a poor collembolan community concerning density and species number. 

Most millipede and centipede species are not well adapted to regular and aperiodic flood events. Some species show a “risk strategy” with a combination of high reproduction rates, dispersal and reimmigration following flood events. Thus only a few opportunistic species are able to inhabit the riparian zones and floodplain forests of Central European river habitats. The bioindication value of these species is lower than that of springtails, because these species occur in frequently inundated sites as well as in non-flooded sites. The predicted climate change could result in smaller effects for the myriapod community. Many species are well adapted to drought conditions. Long-lasting drought events will be survived curled in deeper soil layers [122]. Therefore the species composition of the different riparian zones and floodplain forests will not change basically, but changes in the abundance of some species are possible.

Spiders are in contrast to springtails, millipedes and centipedes more mobile and can therefore change habitats which offer unfavorable conditions. Spiders are considered as an arthropod group with very high bioindication level, which is illustrated in their use in many different plan-approval procedures. Central European riparian zones and floodplain forests are inhabited by a good number of stenotopic spider species. They are well adapted to regular and aperiodic flood events. Extreme and long-lasting drought events cause in strong decreasing densities and finally in the disappearance of the stenotopic species. They will be replaced by more ubiqituous and opportunistic species. Such an effect was observed after the extreme hot and dry summer 2003 in the Northern Upper Rhine Valley. However, the use of stenotopic riparian species as indicators to estimate flood intensity is difficult. Their distribution in regularly flooded regions is not consistent, since the range of stenotopic species varies greatly depending on geographic location (pers. comm. Z. Krumpálová). Furthermore due to the high mobility of many species yearly changes of the species composition and density are possible. Thus the use of spiders as single bioindicator group is not sufficient for the classification of riparian sites in Central Europe. 

The effects of regional climate change with aperiodic flooding and prolonged drought events on the above mentioned arthropod coenoses can still not be estimated in total. However, it can be assumed that some riparian zone specialist species will be permanently lost because of changed flood dynamics, although opportunistic and euryoecious species may stand a better chance of survival. These observations were made in floodplain forests and polder sites of the Northern Upper Rhine Valley after the extreme summer drought of 2003 with an absence of regular flooding for eight years until spring 2012.

## 6. Conclusions

In summary, in the Central European floodplain forests and wetlands, just as in the Amazonian floodplain forests, there are many different adaptations and predispositions for survival of periodic flooding and drought in a number of different arthropod taxa. Mainly the different phenological and morphological predispositions are very valuable to survive periodic flooding and normal drought events in the floodplain habitats. But aperiodic flooding and extreme drought events are of outstanding influence for the arthropod community of riparian habitats. A few stenotopic species bear adaptations to survive these conditions, but many well adapted floodplain species show markedly decreases in abundance and disappear after long-term disturbance by these events. In the future, the consequences of such species depletion in sensitive floodplain forests, riparian sites and wetlands should be a major focus in the programs of Central European floodplain research.

## References

[1] Adis J. (1992). Überlebensstrategien terrestrischer Invertebraten in Überschwemmungswäldern Zentralamazoniens. Verhandlungen des Naturwissenschaftlichen Vereins Hamburg (NF).

[2] Adis J., Junk W.J. (2002). Terrestrial invertebrates inhabiting lowland river floodplains of Central Amazonia and Central Europe: A review. Freshw. Biol..

[3] Junk W.J. (1997). Ecological Studies 126—The Central Amazon Floodplain. Ecology of a Pulsing System.

[4] Junk W.J., Nunes da Cunha N., Wantzen K.M., Petermann P., Strüssmann C., Marques M.I., Adis J. (2006). Biodiversity and its conservation in the Pantanal of Mato Grosso, Brazil. Aquat. Sci..

[5] Weigmann G., Wohlgemuth-von Reiche D., Dohle W., Bornkamm R., Weigmann G. (1999). Vergleichende Betrachtungen zu den Bodentieren im Überflutungsbereich von Tieflandauen. Limnologie Aktuell Band 9, Das Untere Odertal.

[6] Deharveng L., D’Haese C.A., Bedos A. (2008). Global diversity of springtails (Collembola; Hexapoda) in freshwater. Hydrobiologia.

[7] Deharveng L., Lek S. (1995). High diversity and community permeability: The riparian Collembola (Insecta) of a *Pyrenean massif*. Hydrobiologia.

[8] Hering D., Gerhard M., Manderbach R., Reich M. (2004). Impact of a 100-year flood on vegetation, benthic invertebrates, riparian fauna and large woody debris standing stock in an alpine floodplai. River Res. Appl..

[9] Marx M.T., Wild A.-K., Knollmann U., Kamp G., Wegener G., Eisenbeis G. (2009). Responses and adaptations of collembolan communities (Hexapoda: Collembola) to flooding and hypoxic conditions. Pesquisa Agropecuária Brasileira.

[10] Plum N. (2005). Terrestrial invertebrates in flooded grassland: A literature review. Wetlands.

[11] Rothenbücher J., Schaefer M. (2005). Conservation of leafhoppers in floodplain grasslands—Trade-off between diversity and naturalness in a northern German national park. J. Insect Conserv..

[12] Rothenbücher J., Schaefer M. (2006). Submersion tolerance in floodplain arthropod communities. Basic Appl. Ecol..

[13] Tamm J.C. (1982). Das jahresperiodisch trockenliegende Eulitoral der Edertalsperre als Lebens-und Ersatzlebensraum; eine Ökosystemstudie mit terrestrischem Schwerpunkt. Arch. Hydrobiol. Suppl. Algol. Stud..

[14] Tamm J.C. (1984). Surviving long submergence in the egg stage—A successful strategy of terrestrial arthropods living on floodplains (Collembola, Acari, Diptera). Oecologia.

[15] Tamm J.C. (1986). Temperature-controlled under-water egg dormancy and post-flood hatching in *Isotoma viridis* (Collembola) as forms of adaptation to annual long-term flooding. Oecologia.

[16] Tamm J.C., Mittmann H.W., Woas S. (1984). Zur Landmilbenfauna eines jahresperiodisch trockenfallenden Stauseebodens. Pedobiologia.

[17] Jucevica E., Melecis V. (2005). Global warming affect Collembola community: A long-term study. Pedobiologia.

[18] Archaux F., Wolters V. (2006). Impact of summer drought on forest biodiversity: What do we know?. Ann. For. Sci..

[19] McMahon T.A., Finlayson B.L. (2003). Droughts and anti-droughts: The low-flow hydrology of Australian rivers. Freshw. Biol..

[20] Humphries P., Baldwin D.S. (2003). Drought and aquatic ecosystems: An introduction. Freshw. Biol..

[21] Kundzewicz Z.W., Ulbrich U., Brücher T., Graczyk D., Krüger A., Leckebusch G.C., Menzel L., Pinskwar I., Radziejewski M., Szwed M. (2005). Summer floods in Central Europe—Climate Change Track?. Natural Hazards.

[22] Christensen O.B., Goodess C.M., Ciscar J.-C. (2012). Methodological framework of the PESETA project on the impacts of climate change in Europe. Clim. Change.

[23] Jentsch A., Kreyling J., Boettcher-Treschkow J., Beierkuhnlein C. (2009). Beyond gradual warming: Extreme weather events alter flower phenology of European grassland and heath species. Glob. Chang. Biol..

[24] Jöhnk K.D., Huisman J., Sharples J., Sommeijer B., Visser P.M., Stroom J.M. (2008). Summer heatwaves promote blooms of harmful cyanobacteria. Glob. Chang. Biol..

[25] Kyselý J., Gaál L., Beranová R., Plavcová E. (2011). Climate change scenarios of precipitation extremes in Central Europe from ENSEMBLES regional climate models. Theor. Appl. Climatol..

[26] Milad M., Schaich H., Bürgi M., Konold B. (2011). Climate change and nature conservation in Central European forests: A review of consequences, concepts and challenges. For. Ecol. Manage..

[27] Nikulin G., Kjellström E., Hansson U., Strandberg G., Ullerstig A. (2011). Evaluation and future projections of temperature, precipitation and wind extremes over Europe in an ensemble of regional climate simulations. Tellus (A).

[28] Schär C., Vidale P.L., Lüthi D., Frei C., Häberli C., Liniger M.A., Appenzeller C. (2004). The role of increasing temperature variability in European summer heatwaves. Nature.

[29] Verzano K., Bärlund I., Flörke M., Lehner B., Kynast E., Voß F., Alcamo J.  (2012). Modeling variable river flow velocity on continental scale: Current situation and climate change impacts in Europe. J. Hydrol..

[30] Luterbacher J., Dietrich D., Xoplaki E., Grosjean M., Wanner H. (2004). European seasonal and annual variability, trends and extremes since 1500. Science.

[31] Schindler U., Steidl J., Müller L., Eulenstein F., Thiere J. (2007). Drought risk to agricultural land in Northeast and Central Germany. J. Plant Nutr. Soil Sci..

[32] (2007). Das Abflussregime des Rheins und seiner Nebenflüsse im 20. Jahrhundert—Analyse, Veränderungen, Trends; Final Report.

[33] Klein Tank A.M.G., Können P.G. (2003). Trends in indices of daily temperature and precipitation extremes in Europe, 1946–99. J. Clim..

[34] Moberg A., Jones P.D. (2005). Trends in indices for extremes in daily temperature and precipitation in Central and Western Europe, 1901–99. Int. J. Climatol..

[35] Schröter D., Zebisch M., Grothmann T. (2005). Climate Change in Germany—Vulnerability and Adaptation of Climate-Sensitive Sectors; Annual Report.

[36] Molnar P., Favre V., Perona P., Burlando P., Randin C., Ruf W. (2008). Floodplain forest dynamics in a hydrologically altered mountain river. Peckiana.

[37] Hirst S., Maulik S. (1926). On some arthropod remains from the Rhynie chert (old red sandstone). Geol. Mag..

[38] Whalley P., Jarzembowski E.A. (1981). A new assessment of *Rhyniella*, the earliest known insect, from the Devonian of Rhynie, Scotland. Nature.

[39] Habgood K.S., Hass H., Kerp H. (2004). Evidence for an early terrestrial food web: Coprolites from the early Devonian Rhynie chert. Trans. R. Soc. Edinb. Earth Sci..

[40] Bauer R., Christian E. (1993). Adaptations of three springtail species to granite boulder habitats (Collembola). Pedobiologia.

[41] Brand R.H. (2002). The effect of prescribed burning on epigeic springtails (Insecta: Collembola) of woodland litter. Am. Midl. Nat..

[42] Elnitsky M.A., Benoit J.B., Denlinger D.L., Lee R.E. (2008). Desiccation tolerance and drought acclimation in the Antarctic collembolan *Cryptopygus antarcticus*. J. Insect Physiol..

[43] Greenslade P. (1981). Survival of Collembola in arid environments: Observations in South Australia and the Sudan. J. Arid Environ..

[44] Hawes T.C., Couldridge C.E., Bale J.S., Worland M.R., Convey P. (2006). Habitat temperature and the temporal scaling of cold hardening in the high Arctic collembolan, *Hypogastrura tullbergi* (Schäffer). Ecol. Entomol..

[45] Hawes T.C., Worland M.R., Convey P., Bale J.S. (2007). Aerial dispersal of springtails on the Antarctic Peninsula: Implications for local distribution and demography. Antarct. Sci..

[46] Palissa A., Schwoerbel J., Zwick P. (2000). Collembola. Süßwasserfauna von Mitteleuropa 10.

[47] Shaw P.C.A., Ozanne C., Speight M., Palmer I. (2007). Edge effects and arboreal Collembola in coniferous plantations. Pedobiologia.

[48] Rusek J. (1998). Biodiversity of Collembola and their functional role in the ecosystem. Biodivers. Conserv..

[49] Hopkin S.P. (1997). Biology of the Springtails.

[50] Russell D.J., Schick H., Nährig D., Broll G., Merbach W., Pfeiffer E.M. (2002). Reactions of Soil Collembolan Communities to Inundation in Floodplain Ecosystems of the Upper Rhine Valley. Wetlands in Central Europe.

[51] Griegel A., Dohle W., Bornkamm R., Weigmann G. (1999). Räumliche Verteilung und jahreszeitliche Dynamik von Kleinarthropoden (Collembola, Gamasida) in den Auen des Unteren Odertals. Limnologie Aktuell Band 9, Das Untere Odertal.

[52] Griegel A. (2000). Auswirkungen von Überflutungen auf die Zönosen der Collembolen und der Gamasiden (Insecta: Collembola, Acari: Gamasida) in der Flußaue des unteren Odertals.

[53] Lessel T., Marx M.T., Eisenbeis G. (2011). Effects of ecological flooding on the temporal and spatial dynamics of carabid beetles (Coleoptera: Carabidae) and springtails (Collembola) in a polder habitat. ZooKeys.

[54] Marx M.T. (2008). The collembolan population of a river bank reinforcement system in front of a middle Rhine region floodplain under influence of inundation and extreme drought. Peckiana.

[55] Russell D.J., Griegel A. (2006). Influence of variable inundation regimes on soil Collembola. Pedobiologia.

[56] Russell D.J. (2008). Metacommunity responses of soil Collembola to inundation intensity in the Upper Rhine Valley. Peckiana.

[57] Sterzyńska M., Ehrnsberger R. Diversity and structure of collembolan communities in wetlands. Proceedings of the 5th Central European Workshop on Soil Zoology.

[58] Eisenbeis G., Wichard W. (1985). Atlas zur Biologie der Bodenarthropoden.

[59] Helbig R., Nickerl J., Neinhuis C., Werner C. (2011). Smart skin pattern protect springtails. PLoS ONE.

[60] Marx M.T., Messner B. (2012). A general definition of the term “plastron” in terrestrial and aquatic arthropods. Org. Divers. Evol..

[61] Lawrence P.N., Massoud Z. (1973). Cuticle structures in the Collembola (Insecta). Revue d’Écologie et de Biologie de Sol.

[62] Hale W.G., Smith A.L. (1966). Scanning electron microscope studies of cuticular structures in the genus *Onychiurus* (Collembola). Revue d’Écologie et de Biologie de Sol.

[63] Ghiradella H., Radigan W. (1974). Collembolan cuticle: Wax layer and anti-wetting properties. J. Insect Physiol..

[64] Messner B. (1988). Vorschlag für die Neufassung des Begriffes “Plastron” bei den Arthropoden. Deutsche Entomologische Zeitschrift (N.F.).

[65] Coulson S.J., Hodkinson I.D., Webb N.R., Harrison J.A. (2002). Survival of terrestrial soil-dwelling arthropods on and in seawater: Implications for trans-oceanic dispersal. Funct. Ecol..

[66] Moore P.D. (2002). Springboards for springtails. Nature.

[67] Coulson S.J., Birkemoe T. (2000). Long-term cold tolerance in Arctic invertebrates: Recovery after 4 years at below −20 °C. Can. J. Zool..

[68] Fridriksson S. (1975). Surtsey, Evolution of Life on a Volcanic Island.

[69] Zinkler D., Platthaeus J. (1996). Tolerance of soil-dwelling Collembola to high carbon dioxide concentrations. Eur. J. Entomol..

[70] Zinkler D. (1966). Vergleichende Untersuchungen zur Atmungsphysiologie von Collembolen (Apterygota) und anderen Kleinarthropoden. Z. Vgl. Physiol..

[71] Zinkler D., Rüssbeck R. Ecophysiological adaptations of Collembola to low oxygen concentrations. Proceedings of 2nd International Seminar on Apterygota.

[72] Paul R.J., Colmorgen M., Hüller S., Tyroller F., Zinkler D. (1997). Circulation and respiration control in millimeter-sized animals (*Daphnia magna*, *Folsomia candida*) studied by optical methods. J. Comp. Physiol. B.

[73] Zinkler D., Rüssbeck R., Biefang M., Baumgärtl H. (1999). Intertidal respiration of *Anurida maritima* (Collembola: Neanuridae). Eur. J. Entomol..

[74] Joosse E.N.G. (1966). Some observations on the biology of *Anurida maritima* (Collembola). Zeitschrift für Morphologie und Ökologie der Tiere.

[75] Blancquaert J.P., Coessens R., Mertens J. (1981). Life history of some Symphypleona (Collembola) under experimental conditions. I. Embryonal development and Diapause. Revue d’Écologie et de Biologie de Sol.

[76] Gauer U. (1997). Collembola in Central Amazon inundation forests—Strategies for surviving floods. Pedobiologia.

[77] Thibaud J.M. (1970). Biologie et écologie des Collemboles Hypogastruridae édaphiques et cavernicoles. Mémoires du Muséum National d‘Histoire Naturelle A.

[78] Beck L. (1972). Der Einfluss der jahresperiodischen Überflutungen auf den Massenwechsel der Bodenarthropoden im zentralamazonischen Regenwaldgebiet. Pedobiologia.

[79] Alvarez T., Frampton G.K., Goulson D. (1999). The effects of drought upon epigeal Collembola from arable soils. Agric. For. Entomol..

[80] Lindberg N., Bengtsson J. (2005). Population responses of oribatid mites and collembolans after drought. Appl. Soil Ecol..

[81] Lindberg N., Bengtsson J. (2006). Recovery of forest soil fauna diversity and composition after repeated summer droughts. Oikos.

[82] Meier P., Zettel J. (1997). Cold hardiness in *Entomobrya nivalis* (Collembola, Entomobryidae): Annual cycle of polyols and antifreeze proteins, and antifreeze triggering by temperature and photoperiod. J. Comp. Physiol. B.

[83] Simon H.R. (2007). *Entomobrya nivalis* (Linnaeus, 1758) als dominante Art im Nahrungssystem von Apfelbaumkronen—Zwischenergebnisse aus dem Projekt „Monitoring von Arthropoden in Apfelanlagen“ (Collembola). Entomol. Z. Insektenbörse.

[84] Vegter J.J. (1987). Phenology and seasonal resource partitioning in forest floor Collembola. Oikos.

[85] Massoud Z.N., Poinsot N., Poivre C. (1968). Contribution à l’étude du comportement constructeur chez les Collemboles. Revue d’Écologie et de Biologie de Sol.

[86] Poinsot N. (1970). Nouveaux exemples de comportement constructeur chez les collemboles Isotomidae. Revue du Comportement Animal.

[87] Poinsot N. (1971). Contribution à l’étude du comportement constructeur chez les Collemboles. Revue d’Écologie et de Biologie de Sol.

[88] Belgnaoui S., Barra J.A. (1989). Water loss and survival in anhydrobiotic Collembola *Folsomides angularis* (Insecta). Revue d’Écologie et de Biologie de Sol.

[89] Poinsot-Balaguer N., Barra J.A. (1991). L’anhydrobiose: un problème biologique nouveau chez les Collemboles (Insecta). Revue d’Écologie et de Biologie de Sol.

[90] Block W. (1996). Cold or drought—The lesser of two evils for terrestrial arthropods. Eur. J. Entomol..

[91] Holmstrup M., Hedlund K., Boriss H. (2002). Drought acclimation and lipid composition in *Folsomia candida*: implications for cold shock, heat shock and acute desiccation stress. J. Insect Physiol..

[92] Poinsot-Balaguer N., Barra J.A. (1983). Experimental and ultrastructural data on freezing resistance of *Folsomides angularis* (Insecta, Collembola). Pedobiologia.

[93] Worland M.R., Grubor-Lajsic G., Montiel P.O. (1998). Partial desiccation induced by sub-zero temperatures as a component of the survival strategy of the Arctic collembolan *Onychiurus arcticus* (Tullberg). J. Insect Physiol..

[94] Hinton H.E. (1960). Cryptobiosis in the larva of *Polypedilum vanderplanki* Hint. (Chironomidae). J. Insect Physiol..

[95] Cassagnau P. (1971). Les différents types d’écomorphose chez les collemboles Isotomidae. Revue d’Écologie et de Biologie de Sol.

[96] Fountain M.T., Hopkin S.P. (2005). *Folsomia candida* (Collembola): A “standard” soil arthropod. Annu. Rev. Entomol..

[97] Sjursen H., Bayley M., Holmstrup M. (2001). Enhanced drought tolerance of a soil-dwelling springtail by pre-acclimation to a mild drought stress. J. Insect Physiol..

[98] Pflug A., Wolters V. (2001). Influence of drought and litter age on Collembola communities. Eur. J. Soil Biol..

[99] Sjursen H., Holmstrup M. (2004). Cold and drought stress in combination with pyrene exposure: studies with *Protaphorura armata* (Collembola: Onychiuridae). Ecotoxicol. Environ. Saf..

[100] Kaersgaard C.W., Holmstrup M., Malte H., Bayley M. (2004). The importance of cuticular permeability, osmolyte production and body size for the desiccation resistance of nine species of Collembola. J. Insect Physiol..

[101] Bayley M., Holmstrup M. (1999). Water vapour absorption in arthropods by accumulation of myoinositol and glucose. Science.

[102] Duffey E. (1966). Spider ecology and habitat structure (Arach., Araneae). Senckenb. Biol..

[103] Foelix R.F. (1996). Biology of Spiders.

[104] Beyer W., Grube R. (1997). Einfluss des Überflutungsregimes auf die epigäische Spinnen-und Laufkäferfauna an Uferabschnitten im Nationalpark “Unteres Odertal”. Verhandlungen der Gesellschaft für Ökologie.

[105] Bonn A., Hagen K., Wohlgemuth-von Reiche D. (2002). The significance of flood regimes for carabid beetle and spider communities in riparian habitats—A comparison of tree major rivers in Germany. River Res. Appl..

[106] Thaler K., Pintar M., Steiner H.M. (1984). Fallenfänge von Spinnen in den östlichen Donauauen (Stockerau, Niederösterreich). Spixiana.

[107] Siepe A. (1985). Einfluss häufiger Überflutungen auf die Spinnen-Besiedlung am Oberrhein-Ufer. Mitt. Dtsch. Ges. Allg. Angew. Ent..

[108] Wohlgemuth-von Reiche D., Grube R., Dohle W., Bornkamm R., Weigmann G. (1999). Zur Lebensraumbindung der Laufkäfer und Webspinnen (Coleoptera, Carabidae; Araneae) im Überflutungsbereich der Odertal-Auen. Limnologie Aktuell Band 9, Das Untere Odertal.

[109] Richter C.J.J. (1970). Aerial dispersal in relation to habitat in eight wolf spider species (*Pardosa*, Araneae, Lycosidae). Oecologia.

[110] Suter R.B. (1999). An aerial lottery: The physics of ballooning in a chaotic atmosphere. J. Arachnol..

[111] Thomas C.F.G., Jepson P.C. (1999). Differential aerial dispersal of linyphiid spiders from a grass and a cereal field. J. Arachnol..

[112] Kiss B., Samu F. (2002). Comparison of autumn and winter development of two wolf spider species (*Pardosa*, Lycosidae, Araneae) having different life history patterns. J. Arachnol..

[113] Bauchhenss E. (1991). Die epigäische Spinnenfauna eines Auwaldgebietes der Donau im Landkreis Dillingen/Donau (Deutschland, Bayern). Arachnologische Mitteilungen.

[114] Manderbach R. (2001). Der Stellenwert des Lebenszyklus für das Überleben der Ufer bewohnenden Wolfspinnenarten *Pardosa wagleri* (Hahn, 1822) und *Pirata knorri* (Scopoli, 1763. Arachnologische Mitteilungen.

[115] Krumpálová Z. Floods—As the factor of degradation and recovery of araneocoenoses. Proceedings of the 7th Central European Workshop on Soil Zoology.

[116] Kubcová L., Schlaghamerský J. (2002). Zur Spinnenfauna der Stammregion stehenden Totholzes in südmährischen Auenwäldern. Arachnologische Mitteilungen.

[117] Zulka K.P. (1989). Einfluss der Hochwässer auf die epigäische Arthropodenfauna im Überschwemmungsbereich der March (Niederösterreich). Mitt. Dtsch. Ges. Allg. Angew. Ent..

[118] Stratton G.E., Suter R.B., Miller P.R. (2004). Evolution of water surface locomotion by spiders: A comparative approach. Biol. J. Linn. Soc. Lond..

[119] Seymour R.S., Hetz S.K. (2011). The diving bell and the spider: The physical gill of *Argyroneta aquatic*. J. Exp. Biol..

[120] Pétillon J., Montaigne W., Renault D. (2009). Hypoxic coma as a strategy to survive inundation in a salt-marsh inhabiting spider. Biol. Lett..

[121] Steinberger K.H. (2004). Zur Spinnenfauna der Parndorfer Platte, einer Trockenlandschaft im Osten Österreichs (Burgenland) (Arachnida: Araneae, Opiliones). Denisia.

[122] Edney E.B., Farner D.S., Hoar W.S., Hoelldobler B., Langer H., Lindauer M. (1977). Water balance in land arthropods. Zoophysiology and Ecology.

[123] Barth F.G. (1969). Die Feinstruktur des Spinneninteguments. 1. Die Cuticula des Laufbeins adulter häutungsferner Tiere (*Cupiennius salei*, Keys.). Z. Zellforsch. Mikrosk. Anat..

[124] Barth F.G. (1970). Die Feinstruktur des Spinneninteguments. II. Die räumliche Anordnung der Mikrofasern in der lamellierten Cuticula und ihre Beziehung zur Gestalt der Porenkanäle (*Cupiennius salei*, Keys., adult, häutungsfern, Tarsus). Z. Zellforsch. Mikrosk. Anat..

[125] Hadley N.F., Quinlan M.C. (1989). Cuticular permeability of the black widow spider *Latrodectus hesperus*. J. Comp. Physiol. B.

[126] Hadley N.F., Ahearn G.A., Howarth G. (1981). Water and metabolic relations of cave-adapted and epigean lycosid spiders in Hawaii. J. Arachnol..

[127] Humphreys W.F. (1975). The influence of burrowing and thermoregulatory behaviour on the water relations of *Geolycosa godeffroyi* (Araneae: Lycosidae), an Australian wolf spider. Oecologia.

[128] Ehn R., Tichy H. (1994). Hygro- and thermoreceptive tarsal organ in the spider *Cupiennius salei*. J. Comp. Physiol. A.

[129] Tichy H., Loftus R. (1996). Hygroreceptors in insects and a spider: Humidity Transduction Models. Naturwissenschaften.

[130] Machin J., Lampert G.J. (1985). A passive two layer permeability-water model for *Periplaneta* cuticle. J. Exp. Biol..

[131] Opell B.D. (1998). The respiratory complementarity of spider book lung and tracheal systems. J. Morphol..

[132] Cloudsley-Thompson J. (1957). Nocturnal ecology and water regulation of British cribellate spiders of the genus *Ciniflo*. Biol. J. Linn. Soc. Lond..

[133] Levi H.W. (1967). Adaptations of respiratory systems of spiders. Evolution.

[134] Levi H.W., Kirber W.M. (1976). On the evolution of tracheae in arachnids. Bull. Br. Arachnol. Soc..

[135] Finke T., Paul R. (1989). Book lung function in arachnids III. The function of the spiracles. J. Comp. Physiol. B.

[136] Maddrell S.H.P. (1981). The functional design of the insect excretory system. J. Exp. Biol..

[137] Parry D.A. (1954). On the drinking of soil capillary water by spiders. J. Exp. Biol..

[138] Butt A.G., Taylor H.H. (1995). Regulatory responses of the coxal organs and the anal excretory system to dehydration and feeding in the spider *Porrhothele antipodiana* (Mygalomorha: Dipluridae). J. Exp. Biol..

[139] Hieber C.S. (1992). The role of spider cocoons in controlling desiccation. Oecologia.

[140] Austin A.D. (1984). Life history of *Clubiona robusta* L. Koch and related species (Araneae: Clubionidae) in South Australia. J. Arachnol..

[141] Schaefer M. (1976). An analysis of diapause and resistance in the egg stage of *Floronia bucculenta* (Araneae: Linyphiidae). Oecologia.

[142] Austin A.D., Anderson D.T. (1978). Reproduction and development of the spider *Nephila edulis* (Koch) (Araneae: Araneidae). Aust. J. Zool..

[143] Humphreys W.F. (1991). Thermal behaviour of a small spider (Araneae: Araneidae: Araneinae) on horizontal webs in semi-arid Western Australia. Behav. Ecol. Sociobiol..

[144] Adis J. (1986). An “aquatic” millipede from a Central Amazonian inundation forest. Oecologia.

[145] Adis J. (1992). On the survival strategy of *Mestosoma hylaeicum* Jeekel, a millipede from central Amazonian floodplains (Paradoxosomatidae, Polydesmida, Diplopoda). Berichte des naturwissenschaftlich-medizinischen Vereins in Innsbruck. Supplementum.

[146] Zerm M., Dohle W., Bornkamm R., Weigmann G. (1999). Vorkommen und Verteilung von Tausendfüßern, Hundertfüßern, Zwergfüßern (Myriapoda: Diplopoda, Chilopoda, Symphyla) und Landasseln (Isopoda: Oniscidea) in den Auen des Unteren Odertals. Limnologie Aktuell Band 9, Das Untere Odertal.

[147] Zerm M. (1997). Distribution and phenology of *Lamyctes fulvicornis* and other lithobiomorph centipedes in the floodplain of the Lower Oder Valley, Germany (Chilopoda: Henicopidae, Lithobiidae. Entomol. Scand. Suppl..

[148] Zulka K.P. (1991). Überflutung als ökologischer Faktor Verteilung, Phänologie und Anpassung der Diplopoda, Lithobiomorpha und Isopoda in den Flußauen der March. Ph.D. Thesis.

[149] Tufova J., Tuf I.H., Tajovský K., Schlaghamerský J., Pižl V. (2005). Survival under water—Comparative study of millipedes (Diplopoda), centipedes (Chilopoda) and terrestrial isopods (Oniscidea). Proceedings of the 7th Central European Workshop on Soil Zoology, České Budějovice, Czech Republic, 2003.

[150] Verhoeff K.W. (1926). Vom Einflusse unbewegten Wassers auf Tausendfüßler. 104. Diplopoden-Aufsatz. Zool. Anz..

[151] Schubart O. (1934). Tausendfüßler oder Myriapoda. I: Diplopoda. Die Tierwelt Deutschlands und der angrenzenden Meeresteile.

[152] Thiele H.U. (1968). Die Diplopoden des Rheinlandes. Decheniana.

[153] Eason E.H. (1964). The Centipedes of the British Isles.

[154] Keay A.N., Forman R.I. (1987). An experimental study of the tolerance of *Hapolophilus subterraneus* (Shaw) and *Henia vesuviana* (Newport) to low humidity levels. Bull. British Myriapod Group.

[155] Hopkin S.P., Read H.J. (1992). The Biology of Millipedes.

[156] Krishnan G. (1968). The millipede *Thyropygus* with special reference to Indian species. CSIR Zool. Mem. Indian Anim. Types.

[157] Blower J.G., Kevan D.K.M. (1955). Millipedes and centipedes as soil animals. Soil Zoology.

[158] Demange J.M., Mauriès J.P. (1975). Données de morphologie, tératologie, développement postembryonnaire, faunistique et écologie des Myriapodes Diplopodes nuisibles aux cultures du Sénégal. Bulletin du Muséum national d'histoire naturelle, 3eme série, Zoologie.

[159] Haacker U. (1968). Deskriptive, experimentelle und vergleichende Untersuchungen zur Autökologie rhein-mainischer Diplopoden. Oecologia.

[160] Lewis J.G.E. (1974). The ecology of centipedes and millipedes in Northern Nigeria. Symp. Zool. Soc. London.

[161] Crawford C.S., Camatini M. (1979). Desert millipedes a rationale for their distribution. Myriapod Biology.

[162] Crawford C.S., Matlack M.C. (1979). Water relations of desert millipede larvae, larva-containing pellets and surrounding soil. Pedobiologia.

[163] Bercovitz K., Warburg M.R. (1988). Factors affecting egg-laying and clutch size of *Archispirostreptus tumuliporus judaicus* (Attems) (Myriapoda), Diplopoda in Israel. Soil Biol. Biochem..

